# Osmolality of Cerebrospinal Fluid from Patients with Idiopathic Intracranial Hypertension (IIH)

**DOI:** 10.1371/journal.pone.0146793

**Published:** 2016-01-25

**Authors:** Elisabeth A. Wibroe, Hanne M. Yri, Rigmor H. Jensen, Morten A. Wibroe, Steffen Hamann

**Affiliations:** 1 Department of Ophthalmology, Rigshospitalet, University of Copenhagen, Glostrup, Denmark; 2 Danish Headache Center, Department of Neurology, Rigshospitalet, University of Copenhagen, Glostrup, Denmark; 3 Department of Pediatrics and Adolescent Medicine and department of Neurosurgery, Rigshospitalet, University of Copenhagen, Copenhagen, Denmark; Istanbul University, TURKEY

## Abstract

**Introduction:**

Idiopathic intracranial hypertension (IIH) is a disorder of increased intracranial fluid pressure (ICP) of unknown etiology. This study aims to investigate osmolality of cerebrospinal fluid (CSF) from patients with IIH.

**Methods:**

We prospectively collected CSF from individuals referred on suspicion of IIH from 2011–2013. Subjects included as patients fulfilled Friedman and Jacobson’s diagnostic criteria for IIH. Individuals in whom intracranial hypertension was refuted were included as controls. Lumbar puncture with ICP measurement was performed at inclusion and repeated for patients after three months of treatment. Osmolality was measured with a Vapor Pressure Osmometer.

**Results:**

We collected 90 CSF samples from 38 newly diagnosed patients and 28 controls. At baseline 27 IIH-samples and at 3 months follow-up 35 IIH-samples were collected from patients. We found no significant differences in osmolality between 1) patients at baseline and controls (*p = 0*. *86*), 2) patients at baseline and after 3 months treatment (*p = 0*.*97*), and 3) patients with normalized pressure after 3 months and their baseline values (*p = 0*.*79*). Osmolality in individuals with normal ICP from 6–25 cmH_2_O (*n = 41*) did not differ significantly from patients with moderately elevated ICP from 26–45 cmH_2_O (*n = 21*) (*p = 0*.*86*) and patients with high ICP from 46–70 cmH_2_O (*n = 4*) (*p = 0*.*32*), respectively. There was no correlation between osmolality and ICP, BMI, age and body height, respectively. Mean CSF osmolality was 270 mmol/kg (± 1 SE, 95% confidence interval 267–272) for both patients and controls.

**Conclusions:**

CSF osmolality was normal in patients with IIH, and there was no relation to treatment, ICP, BMI, age and body height. Mean CSF osmolality was 270 mmol/kg and constitutes a reference for future studies. Changes in CSF osmolality are not responsible for development of IIH. Other underlying pathophysiological mechanisms must be searched.

## Introduction

Idiopathic intracranial hypertension (IIH) is a relatively rare condition with increased intracranial pressure (ICP) of unknown etiology [1, 2]. Even though visual prognosis is good under sufficient treatment, many patients suffer from severe, chronic headache, and decreased quality of life is known to occur [3–5]. [1, 6].

Overweight and recent weight gain is associated with occurrence of IIH [7–11]. Recent studies also show that childhood overweight or obesity increases the risk of IIH recurrence fivefold [12].

Proposed mechanisms behind IIH include brain parenchymal edema, increased cerebral blood volume, excessive CSF production, venous outflow obstruction and compromised CSF resorption. In recent years, more details about epidemiology and phenotypical presentation have been published along with a proposal of possible contribution of inflammatory factors [13–21]. For a review on analyses of CSF composition in patients with IIH, see Baykan [13]. In this study we investigated the possibility of excess CSF production. It has been shown that overweight is associated with changes in protein composition of the cerebrospinal fluid (CSF), as some proteins were shown to be differentially expressed in the CSF of overweight patients [22]. Thus CSF composition might be changed in patients with IIH. As various CSF components, such as sodium and proteins, are osmotically active, an altered CSF composition would be indicated by changes in CSF osmolality. It is an emerging hypothesis that derangements of the hypothalamic-pituitary-adrenal axis may lead to the development of elevated ICP in IIH via activation of mineralocorticoid receptors in the choroid plexus epithelial cells and subsequent increase of CSF secretion into the ventricles [15, 23, 24]. Fat is an active endocrine tissue that secretes mineralocorticoid-releasing factors, providing a possible link to elevated ICP in overweight patients by an alteration of the renin-aldosterone axis and subsequent increased secretion of sodium into the CSF. In addition increased body height might be associated with higher CSF pressure, although the underlying mechanisms are unknown [25].

Investigating CSF osmolality in patients with IIH could provide valuable knowledge on mechanisms of fluid transport possibly involved in the underlying pathophysiology. We hypothesized that CSF osmolality is changed in IIH, and that the osmolality normalizes after treatment and weight loss. Therefore we aimed to investigate CSF osmolality in patients with IIH and to establish a reference value for CSF osmolality.

## Materials and Methods

### Ethics statement

The present study is a prospective case-control study performed as part of a larger study approved by The National Committee on Health Research Ethics in Denmark. All participants gave informed written consent.

### Inclusion and exclusion criteria

Individuals referred to the Danish Headache Center, Department of Neurology and Department of Ophthalmology at Glostrup Hospital from 2011–2013 suspected of having IIH were recruited.

Patients that fulfilled Friedman and Jacobson’s diagnostic criteria for IIH from 2002 were included as cases [1, 2, 26, 27]. Only patients with newly diagnosed IIH or with relapse after full remission were included, and ICP > 25 cmH_2_O was required. Subjects in whom intracranial hypertension was refuted were included as controls. The control group had ICP ≤ 25 cmH_2_O and no neuro-ophthalmological signs of IIH. All included subjects were aged 15–65 years and had no other known somatic disease except for one control with diabetes.

### Patient history and physical examination

We assessed a detailed patient history in all participants, including prior medical history and exposures, detailed information of current and prior headaches, visual disturbances and other symptoms. All subjects underwent a complete somatic and neurological examination and diagnostic neuroimaging. In all patients with elevated ICP, a CT and/or MRI with venous sequences were performed. All patients, and those of the controls in which papilledema could not be excluded by indirect ophthalmoscopy, underwent a thorough standardized neuro-ophthalmological examination including visual acuity (Snellen), color-vision (Ishihara), visual field autoperimetry (Humphrey 30–2), slit lamp examination, examination of motility, pupillary examination and fundus photo. Body height was noted at time of inclusion. Body weight was measured at baseline and in patients additionally at the 3-month follow-up.

### ICP

All participants had a lumbar puncture (LP) performed at baseline. Opening pressure was measured in a standardized manner with the patient in recumbent position and with stretched legs. Opening pressure was considered a proxy for the ICP. Lumbar puncture and ICP measurement was repeated in patients with IIH after 3 months of treatment.

### Treatment

Weight loss was encouraged in all patients with IIH, and dietary assistance was offered. Treatment with acetazolamide 1000–2225 mg/day and/or topiramate 50–150 mg/day was initiated. Doses depended on the patient’s clinical presentation.

### CSF preparation

We centrifuged collected CSF samples at 2500 rpm for 10 minutes at 4 degrees Celsius. Serum and CSF was pipetted in to Nunc glasses and cooled to minus 80 degrees Celsius immediately after for storage. We measured CSF osmolality using a Vapor Pressure Osmometer (VAPRO model 5600, Triolab). The osmometer was stored in a dry room with a steady room temperature as recommended by the manufacturer. Calibration was carried out as per instructions. For all samples, we performed five consecutive measurements of different 10 μL portions of the same CSF. The mean was noted as the osmolality of the sample. All samples were kept at minus 80 degrees Celsius until use and were kept at 0 degrees Celsius in between measurements. A sample was tilted at least 5 times before a new portion of 10 μL was made to ensure even distribution of solutes in the sample and thereby a more representative portion.

A standardized saline solution of 290 mmol/kg was used to control if the five measurements could be trusted and whenever a measurement was suspect. Measurements were only accepted if the control provided a result from 284–296 mmol/kg, according to the instruction for calibration provided by the fabricant of the osmometer.

In the current substudy we only measured osmolality of CSF, and blood samples were not analyzed.

## Statistics

### Power calculation

To analyze the difference in osmolality between patients and controls, we used 2-sided unpaired t-tests. Assuming that the mean osmolality in the control group is 260 osmol/L, the groups are of equal sizes and the standard deviation is 13, we will with 80% power be able to identify a difference of at least 10 osmol/L at a 5% significance level between the two groups if we include a total of 27 participants in each groups.

### Comparison of patients and controls

Patient and control data were compared using an unpaired t-test. To explore changes during treatment, the follow-up group was subdivided into patients with normalized ICP < 25 cmH_2_O after 3 months of treatment and patients with sustained intracranial hypertension (> 25 cmH_2_O). In patients with normalized ICP after 3 months, baseline and follow-up osmolality was compared by paired t-test.

### ICP Groups

All samples were divided into groups depending on the ICP: “normal” ICP ranging from 6–25 cmH_2_O, “moderately elevated” ICP from 26–45 cmH_2_O and “high” ICP from 46–70 cmH_2_O. In order to avoid paired samples, each participant with paired values was assigned a number. The number would be on a face down paper and drawn to either contribute with a baseline value or a follow-up value for ICP until the baseline and follow-up group consisted of an equal number of participants. An unpaired t-test assuming unequal variance was used to compare the osmolality in the groups.

### BMI Groups

To test whether overweight patients with IIH revealed any changes in osmolality when data were pooled, we divided patient samples into groups according to the World Health Organization BMI classification cutoffs and compared these.

### Correlations

The relationship between osmolality (of all samples) and ICP, BMI, age and body height, respectively, was investigated using Pearson correlation coefficient. Additionally ICP was investigated in relation to BMI and body height.

## Results

### Participants and samples

**Fig 1:**
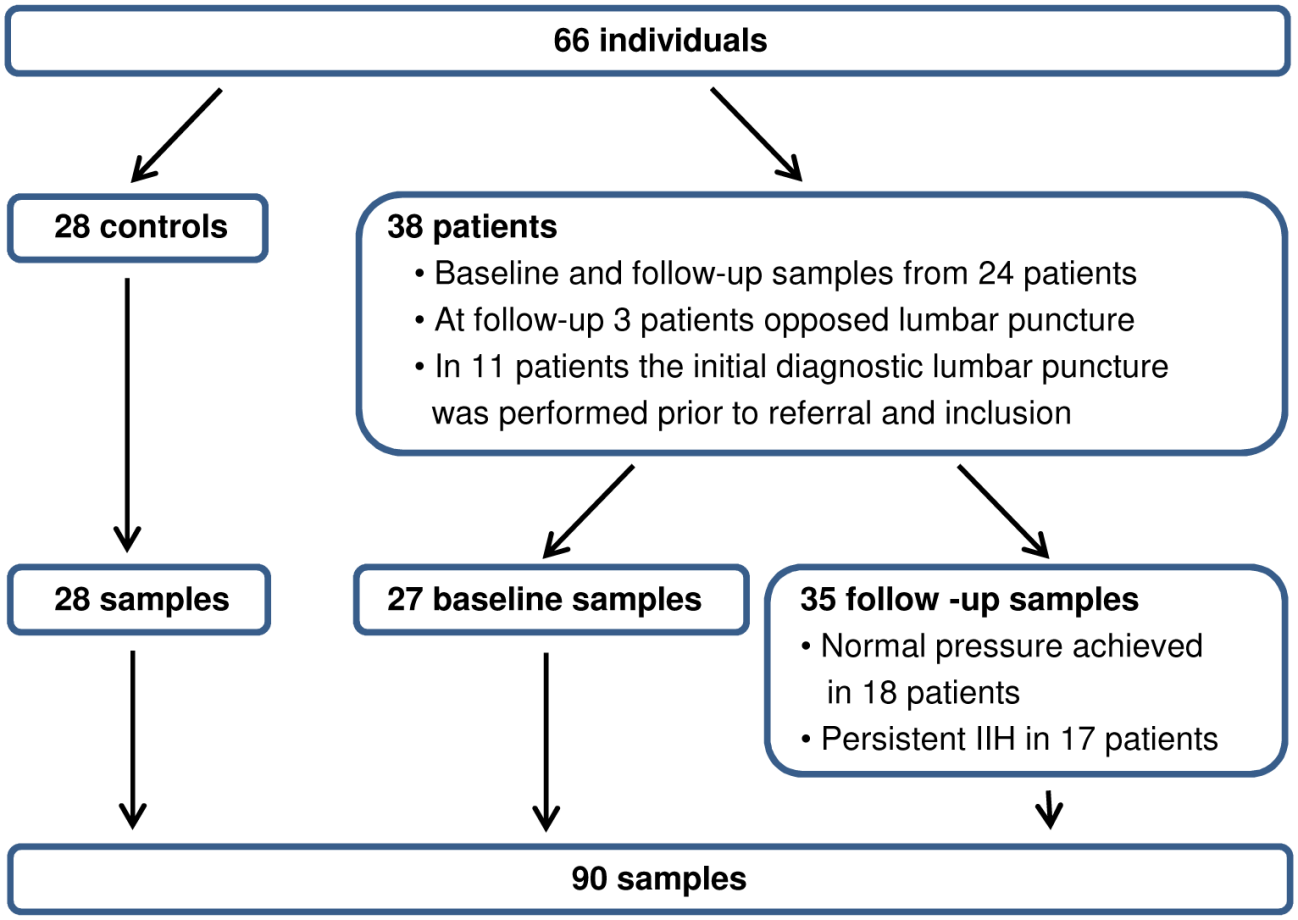
Participants and Samples.

38 patients and 28 controls fulfilled the criteria for participating in the study (Fig 1). Two patients were relapse patients (with at least 10 months without treatment and any symptoms), and 36 were newly diagnosed (included at time of diagnostic lumbar puncture). Four controls had no headache upon referral: one control was referred based on transient visual obscurations, one based on a suspicion of optic disc edema found at a routine control for diabetic retinopathy, one based on significant and pulsating tinnitus and one based on transient visual obscurations, blurry vision and double vision. Otherwise, controls were diagnosed with primary headaches (tension-type, migraine or both). No controls had papilledema. Eight (29%) controls (in addition to all patients with IIH) had a thorough neuro-ophthalmological examination performed to confirm absence of papilledema. Congenital tight or abnormal optic disc morphology was found in 3 of the controls. Ten (36%) controls had ICP between 20–25 cmH2O. All ten were overweight. One patient and two controls were men, and the rest of the participants were women. Most participants were obese according to the World Health Organization BMI classification system with BMI ≥ 30: 85% in the baseline group, 74% in the follow-up group and 60% of the controls. Demographics are presented in Table 1. Paired samples (baseline and follow-up from the same participants) were available from 24 patients.

**Table 1:** Demographics of patients and controls.

	Patients at baseline		Patients at follow-up		Controls	
	*26 female*	*1 male*	*35 female*	*0 male*	*25 female*	*3 male*
	Mean	SE	Mean	SE	Mean	SE
**Age**	31	2	30	2	37	2
**ICP (cmH**_**2**_**O)**	40.4	2	25.2	1	17.7	1
**BMI (kg/m**^**2**^**)**	35	1	34	1	31	1
**Osmolality (mmol/kg)**	269	0	269	0	270	0

### Osmolality

We found no significant differences in osmolality between newly diagnosed patients (*n = 27*, *range*: *232–285 mmol/kg*) and controls (*n = 28*, *range*: *219–283 mmol/kg*) (*p = 0*.*86*). Difference in mean osmolality was 1 mmol/kg. Neither did we find any significant difference between baseline (*range*: *247–285 mmol/kg*) and follow-up (*range*: *246–283 mmol/kg*) in patients with available paired samples (*n = 24*, *difference in mean osmolality*: *4 mmol/kg*, *p = 0*.*32*). Sub-group analysis including only patients with available paired samples and normalized ICP after three months (*n = 10*) similarly showed no significant difference in baseline and follow-up osmolality (*range at baseline*: *247–277 mmol/kg*, *range at follow-up*: *246–283 mmol/kg*, *difference in mean osmolality*: *1 mmol/kg*, *p = 0*.*76*).

**Fig 2:**
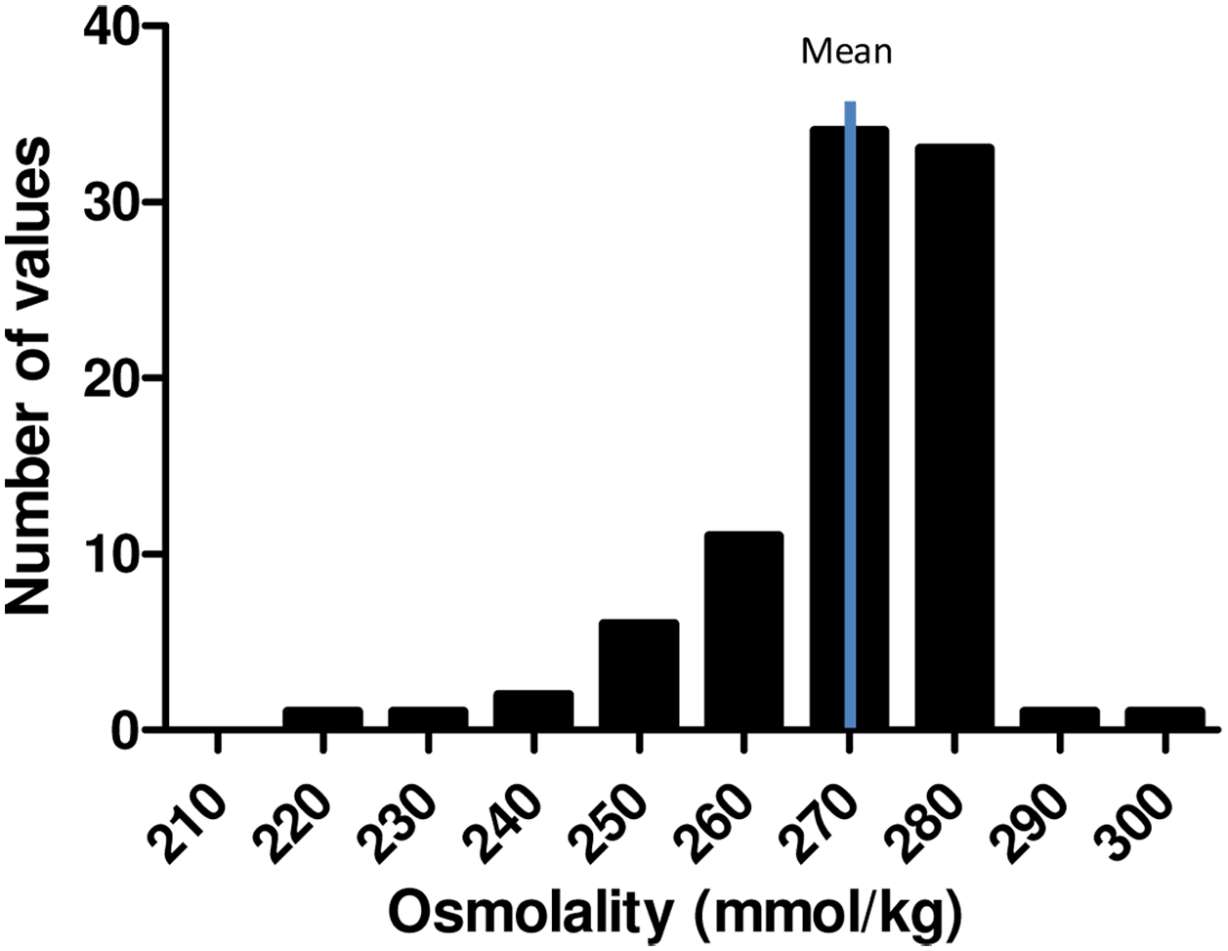
CSF Osmolality Histogram representing Patients and Controls. All 66 participants, 90 samples.

Mean osmolality of all 90 CSF samples was 270 mmol/kg (*± 1 SE*, *95% confidence interval (CI)*: *267–272*) with a range of 219–297 mmol/kg. One control showed a notably low osmolality measuring 219 mmol/kg. Exclusion of this outlier did not affect mean value (mean 270 mmol/kg (± 1 SE)). Twenty-eight percent of all osmolality values were within the 95% CI (Fig 2).

### ICP Groups

**Fig 3:**
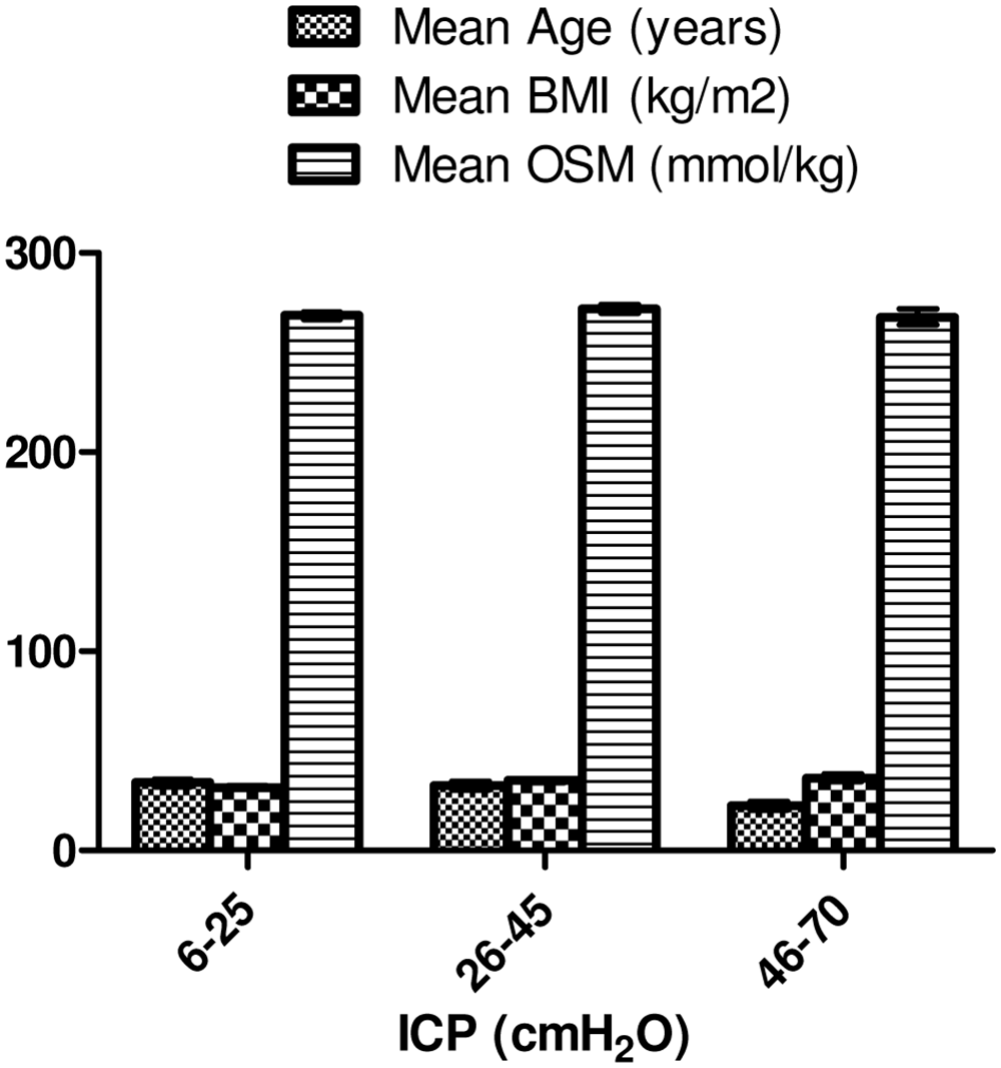
ICP Groups. All 66 participants, 90 samples. Error bars based on SE.

ICP varied from 8–70 cmH_2_O. Patients ranged from 27–70 cmH_2_O at baseline, from 15–38 cmH_2_O at follow-up and controls ranged from 8–25 cmH_2_O (Fig 3). We found no significant differences when comparing osmolality from subjects with “normal” ICP (*n = 41*) with either patients with “moderately elevated” ICP (*n = 21*) (*difference in mean osmolality*: *4 mmol/kg*, *p = 0*.*86*) or patients with “high” ICP (*n = 4*) (*difference in mean osmolality*: *0 mmol/kg*, *p = 0*.*32*).

### BMI Groups

**Fig 4:**
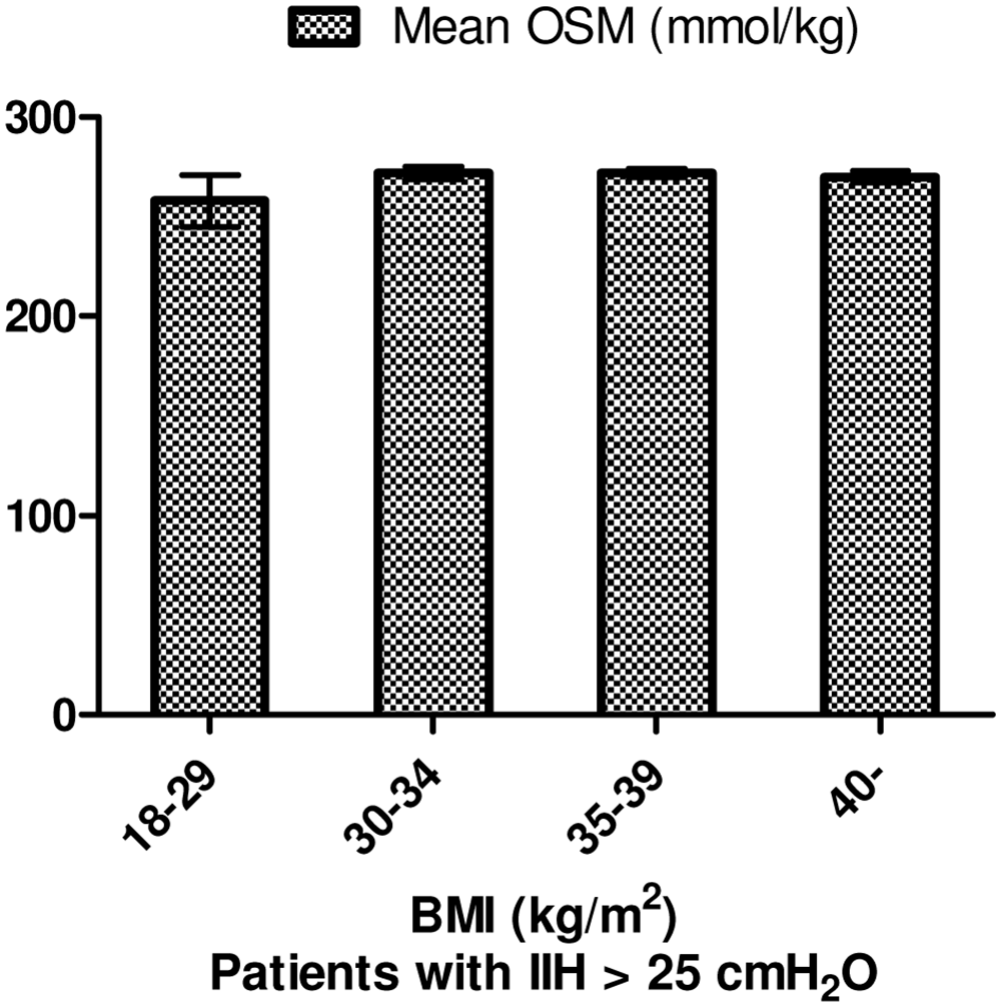
BMI Groups. All 66 participants, 25 unpaired samples. Error bars based on SE.

3 samples were from patients with BMI between 18–29 kg/m^2^, 10 from 30–34 kg/m^2^, 6 from 35–39 kg/m^2^ and 6 from >40 kg/m^2^ after randomization of patients with paired samples (Fig 4). We found no significant difference in osmolality or ICP between the four groups.

### Correlations

We found no correlation when osmolality data from all samples were plotted against ICP, BMI, age and body height, respectively (*R*^*2*^ = *0*.*0041* and *p* = *0*.*55*), (*R*^*2*^ = *0*.*0024* and *p* = *0*.*64*), (*R*^*2*^ = *0*.*0022* and *p* = *0*.*66*), (*R*^*2*^ = *0*.*00027* and *p* = *0*.*88*). ICP was not correlated to BMI or body height (*R*^*2*^ = *0*.*012* and *p* = *0*.*28*), (*R*^*2*^ = *0*.*012* and *p* = *0*.*28*).

## Discussion

Although this study involves almost 100 CSF samples from patients and controls, we were unable to identify any changes in osmolality in IIH patients compared to subjects with normal ICP. Osmolality was neither significantly related to treatment, ICP, BMI, age nor body height.

### Change over Time

Osmolality in patients with IIH did not change over time regardless of normalization of ICP. This indicates that treatment, or simply time, does not change CSF osmolality in patients with IIH, regardless of ICP even when ICP was normalized. This refutes the hypothesis of a reversible change in OSM that normalizes after treatment of IIH.

### ICP Groups

In this study the level of ICP showed no relation to CSF osmolality. Hence, it follows that the level of ICP did not influence results when comparing patient and control osmolality, as not even a considerable pressure difference revealed any osmolality change.

### BMI Groups

Most patients in this study are overweight as in the general IIH population [15]. Salpietro et al. propose that overweight/obesity and IIH are linked by alteration of the renin-aldosterone axis causing an increased secretion of sodium into the CSF via mineralocorticoid receptor activation [23]. However, we did not find any elevation of CSF osmolality in IIH patients, as would be expected if CSF levels of sodium were elevated. Furthermore, our study did not show any direct correlation between BMI per se and level of elevated ICP. The lack of correlation between BMI and ICP could be explained by recent weight gain rather than BMI itself being the triggering factor for IIH [7–11].

### Body Height

A population based study of 3363 subjects at age 50+ years by Jonas et al. suggested that greater body height is associated with a higher CSF pressure [25]. In the present study no such association was found. Jonas et al. used a calculated value for CSF pressure based on BMI, diastolic blood pressure and age, and they adjusted for gender, urban region of habitation, higher educational level and pulse rate. Some of these potential confounders were not accounted for in the present study and may explain the diverging results. Or it might be due to our smaller number of participants. However, considering that osmolality and ICP showed no apparent association, it is reasonable to believe that body height *per se* does not influence osmolality.

### CSF Volume

It has been documented, that the choroid plexus epithelium, despite low overall water permeability, is capable of secreting large amounts of water, probably through a concerted action of the cotransport proteins KCC4 and NKCC1 and the water channel AQP1. These transport mechanisms are all located along with the Na^+^/K^+^ ATPase in the apical cell membrane, for a review see Zeuthen [28]. It is conceivable that these CSF volume regulatory proteins easily overcome small increases in sodium secretion that might happen via an activated Na^+^/K^+^ ATPase. This also provides a likely explanation why ICP of the patients with IIH in the present study can be elevated despite normal CSF osmolality. Thus we do not refute the hypothesis of an altered renin-aldosterone axis behind IIH. Further studies investigating sodium content and protein composition of CSF in patients with IIH are in progress with available blood samples for comparison.

### Mean CSF Osmolality

Based on our results, the mean osmolality in both patients and controls in the present study represents normal CSF osmolality. In a study from 1965 of 50 healthy patients undergoing elective surgery, a reference value of 281 miliosmoles was determined using a Vapor Pressure Osmometer (Mechrolab 301) [29]. In the present study, we provide a mean osmolality of 270 mmol/kg. Given an uncertainty between 2 and 10% considered acceptable by the manufacturer of the equipment used in the present study, the two osmolality values are not far from each other.

Plasma osmolality is normally in the range of 280–300 mmol/kg. Sodium concentration in CSF is slightly higher than in serum [30]. This would provide a higher osmolality in CSF than in serum. However, both our results and older studies provide a lower CSF osmolality than the reference values for plasma osmolality [29]. Thus it should be taken into consideration that CSF osmolality might be lower than plasma osmolality. Further studies on the included patient’s plasma-sodium and plasma osmolality levels at the time of lumbar puncture could provide valuable information on this matter.

### Strengths and limitations

A representative range of well-defined patients with IIH are included in this study, as most patients were overweight, female and newly diagnosed. Friedman and Jacobsen’s classification criteria for IIH diagnosis required ICP > 25 cmH_2_O, and they propose a repeated pressure measurement in patients with a pressure between 20–25 cmH_2_O due to natural fluctuation in CSF pressure [27]. The International Classification of Headache Disorders 2^nd^ edition requires ICP > 25 cmH_2_O in the obese, but only ICP > 20 cmH_2_O in the non-obese, as obese patients normally tend to have a higher ICP [2, 31]. The present study only included patients with ICP > 25 cmH_2_O. Thus this study only included patients with a more certain diagnosis of IIH. Nearly all participants included had headaches upon referral and thus controls were not completely headache free individuals. All, except four, were diagnosed with chronic primary headaches. Including controls with only primary headaches and no suspicion of IIH would address a potential risk of IIH patients accidentally being included as controls. However, diagnosis was not only based on ICP and IIH was further ruled out with detailed history and neuroopthalmological examination. We found that initially suspected of having IIH, the controls were a good match for IIH patients regarding sex, age and BMI, and performing lumbar puncture without clinical indication would have raised ethical concerns.

CSF is a more viscous fluid than the saline solutions constituting the standard solutions for calibration. When we measured osmolality, a little spinal fluid remained in the pipette tip. Therefore, the osmometer was not calibrated to take this into account. Hence, the volume from which the osmometer calculated a given osmolality might have been bigger than the actual volume of the sample, leading to an underestimated value of sample osmolality. However, as this affected both IIH patient and control data, this did not affect comparisons made within and between groups. The true mean values of osmolality might, however, be slightly underestimated.

We provide a mean osmolality based on measurements with the newest equipment in the market. We must, however, remember that an uncertainty between 2 and 10% is considered acceptable by the manufacturer of the equipment.

### Future studies

Definition of IIH has been revised by Friedman et al. in 2013 [26]. Our distinction between patients and controls is in coherence with these criteria. Our results are therefore compatible with future studies that might use the newly revised criteria. Despite the ethical issues of spinal taps in healthy controls, a new control group with healthy, age-, sex- and weight matched individuals could be of interest to include in a future study.

## Conclusion

The study provides valuable knowledge of osmolality in spinal fluid in relation to IIH, ICP, age, BMI, body height and of BMI in relation to ICP in patients with IIH. Neither diagnosis nor treatment of IIH, ICP, age, BMI or height was found to be associated with differences in CSF osmolality. Likewise BMI and body height were found not to be correlated with ICP. Other mechanisms behind IIH than affected CSF osmolality must be searched. In the light of these results, this study provides information on CSF osmolality based on a relatively large cohort with a mean osmolality of 270 mmol/kg (± 1 SE, 95% CI: 267–272), constituting a reference for future studies on CSF osmolality.

## Supporting Information

S1 Dataset Dataset IIH Patients and Controls. (XLSX)

S1 File Symptoms Controls. (XLSX)
